# Building the Perfect Parasite: Cell Division in Apicomplexa 

**DOI:** 10.1371/journal.ppat.0030078

**Published:** 2007-06-29

**Authors:** Boris Striepen, Carly N Jordan, Sarah Reiff, Giel G van Dooren

**Affiliations:** University of British Columbia, Canada

## Abstract

Apicomplexans are pathogens responsible for malaria, toxoplasmosis, and crytposporidiosis in humans, and a wide range of livestock diseases. These unicellular eukaryotes are stealthy invaders, sheltering from the immune response in the cells of their hosts, while at the same time tapping into these cells as source of nutrients. The complexity and beauty of the structures formed during their intracellular development have made apicomplexans the darling of electron microscopists. Dramatic technological progress over the last decade has transformed apicomplexans into respectable genetic model organisms. Extensive genomic resources are now available for many apicomplexan species. At the same time, parasite transfection has enabled researchers to test the function of specific genes through reverse and forward genetic approaches with increasing sophistication. Transfection also introduced the use of fluorescent reporters, opening the field to dynamic real time microscopic observation. Parasite cell biologists have used these tools to take a fresh look at a classic problem: how do apicomplexans build the perfect invasion machine, the zoite, and how is this process fine-tuned to fit the specific niche of each pathogen in this ancient and very diverse group? This work has unearthed a treasure trove of novel structures and mechanisms that are the focus of this review.

## A Lean and Mean Invasion Machine

**Figure 1:**
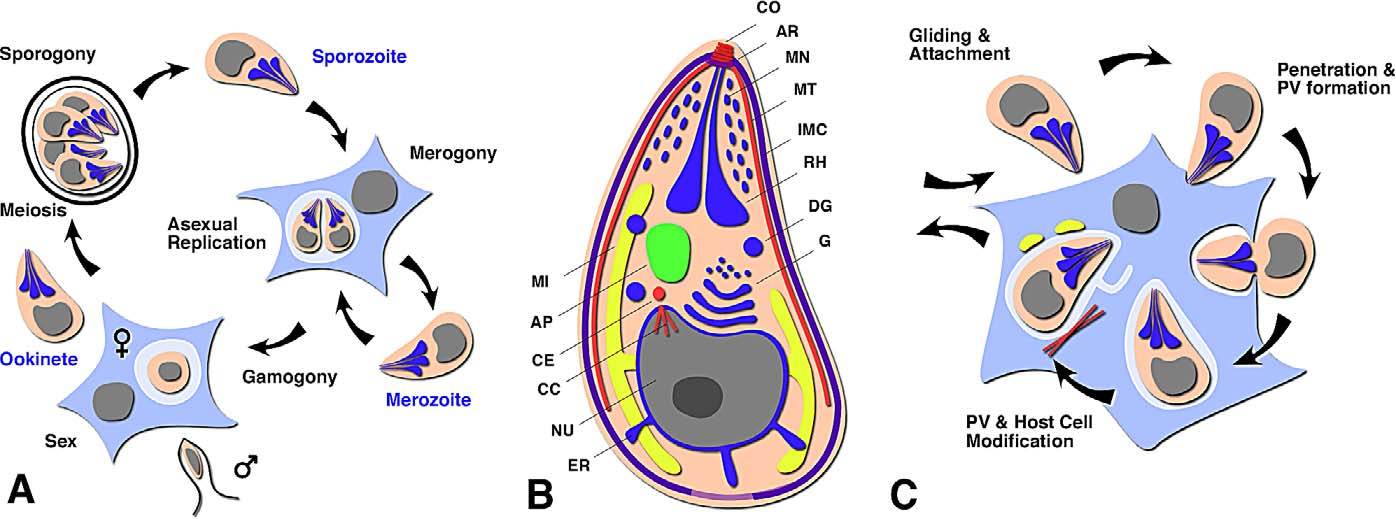
Apicomplexa Are Intracellular Parasites (A) Highly simplified apicomplexan life cycle. Apicomplexans are haplonts, and meiosis (sporogony) immediately follows fertilization. Fertilization might occur within a host cell or extracellularly, giving rise to an oocyst or, less frequently, an invasive stage zygote (ookinete). (B) Schematic representation of a zoite (not all structures are present in all apicomplexans). AP, apicoplast; AR, apical rings; CC, centrocone; CE, centrosome; CO, conoid; DG, dense granule; ER, endoplasmic reticulum; G, Golgi; IMC, inner membrane complex; MI, mitochondrion; MN, microneme; MT, subpellicular microtubule; NU, nucleus; RH, rhoptry. (C) Zoites actively invade the cells of their hosts, establishing a specialized parasitophorous vacuole (PV) (in some species the parasite lyses the vacuole and develops freely in the cytoplasm).

A wide variety of prokaryotic and eukaryotic pathogens have evolved the ability to invade and replicate within the cells of their hosts. Few have developed the level of sophistication and control exerted by the members of the Apicomplexa [1]. Upon contact with a suitable host cell, apicomplexans can invade within seconds, with minimal apparent disturbance of the infected cell (Figure 1). This process is dependent on actin and myosin and is driven by parasite and not host motility [2,3]. Tightly associated with host cell penetration is the secretion of three distinct parasite organelles: rhoptries, micronemes, and dense granules. Secretion is timed in succession, and secreted proteins play key roles in adhesion, motility and formation, and elaboration of the parasitophorous vacuole, a new cellular compartment established during invasion that the parasite occupies during its intracellular development (see [4,5] for detailed reviews of this process in *Toxoplasma* and *Plasmodium,* respectively).

The cellular structure of the zoite, the non-replicative extracellular stage, appears streamlined towards one goal: finding and invading the next host cell. Zoites are found at various stages of the apicomplexan life cycle and are the product of asexual as well as sexual replication processes (see Figure 1A for a simplified apicomplexan life cycle). The zoite is highly polarized, with the apical tip containing the organizing center for the subpellicular microtubles that run along the longitudinal axis of the parasite [6]. This axis also polarizes the cell's motility, driving the parasite into host cells with its apex first. In some species, the tip is further elaborated by the conoid, a cytoskeletal structure that is built from a unique, tightly wound tubulin polymer and is extended during invasion and motility [7]. Importantly, the apical end is also the site for rhoptry and microneme secretion, with these organelles tightly packed into the anterior portion of the cell. While the anterior of the zoite is focused on invasion, the rest of cell carries the genetic material and tools to grow and develop once in the host cell, including a nucleus and a single mitochondrion, plastid, and Golgi.

## Divide and Conquer

**Figure 2:**
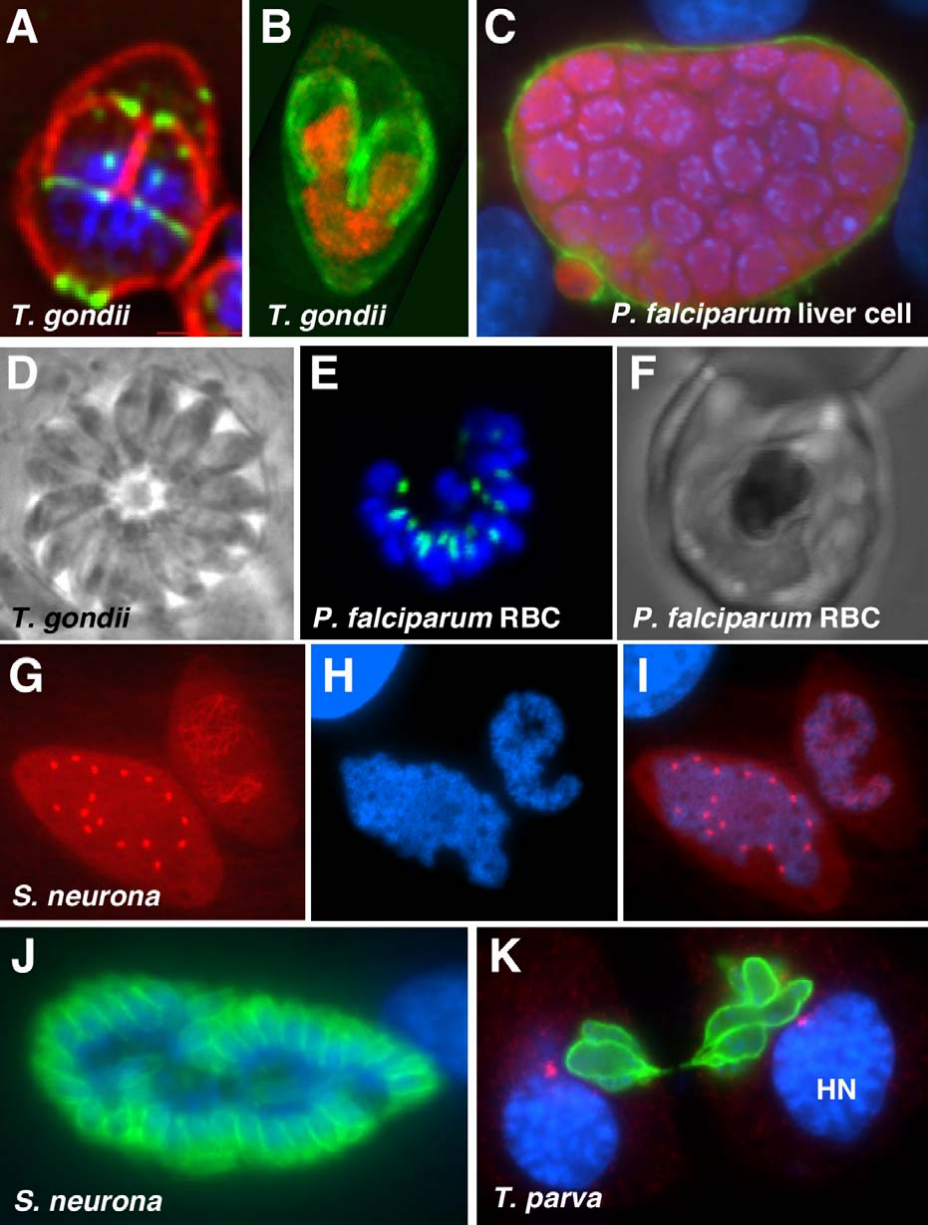
The Diversity of Intracellular Development in Apicomplexans (A) In *T. gondii,* two daughters are formed during budding. IMC1, red; MORN1, green (reproduced with permission from [32]). (B) *T. gondii.* Histone H2, red; IMC3, green (reproduced from [71]). (C) In *Plasmodium falciparum* liver schizont, budding results in massive numbers of zoites. Image courtesy of Volker Heussler. (D) *T. gondii,* phase contrast image of parasitophorous vacuole harboring multiple tachyzoites. (E and F) P. falciparum late erythrocyte schizont. Acyl carrier protein (plastid), green. RBC, red blood cell. (G–I) *Sarcocystis neurona.* Two intracellular stages with polyploid nuclei, one in interphase and one during mitosis. Tubulin, red. (J) S. neurona budding. IMC3, green. (K) A *Theileria* schizont divides in association with its host cell. Polymorphic immunodominant molecule (parasite surface), green; γ-tubulin (host centrosomes), red. HN, host nucleus. Image courtesy of Dirk Dobbelaere. The DNA dye DAPI is shown in blue throughout. Not to scale.

**Figure 3:**
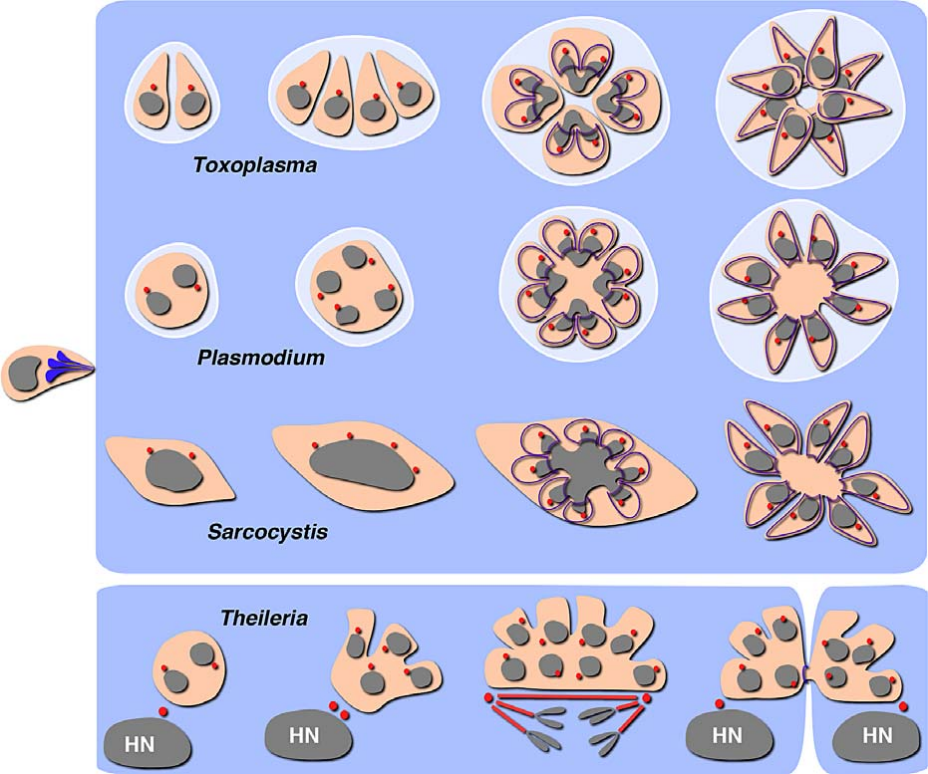
The Flexibility of Apicomplexan Cell Division Schematic outline of cell division by *Toxoplasma* (endodyogeny), *Plasmodium* (schizogony), and *Sarcocystis* (endopolygeny). The *Theileria* schizont is divided in association with host cell division (HN, host nucleus). DNA, grey; IMC, purple; centrosome, red. Note that a centriole as center of the spindle plaque body has not been clearly demonstrated in P. falciparum. Both *Sarcocystis* and *Theileria* develop directly in the host cell cytoplasm, while *Toxoplasma* and *Plasmodium* are contained within a parasitophorous vacuole (light blue).

While invasive zoites are similar across the phylum, intracellular stages differ dramatically in size, shape, and architecture (see Figure 2 for a selection of micrographs). The basis for this diversity lies in the flexibility of the apicomplexan cell cycle. Apicomplexans are able to dissociate and variably mix and match three elements that follow each other invariably in most other cells: DNA replication and chromosome segregation, nuclear division, and, lastly, cytokinesis or budding (see Figure 3 for a schematic). While *Toxoplasma* completes all elements of the cycle after each round of DNA replication, *Plasmodium* and *Sarcocystis* forgo cytokinesis and/or nuclear divisions for multiple cycles, forming stages that are multinucleate or contain a single polyploid nucleus (these division modes are also known as endodyogeny, schizogony, and endopolyogeny [8–10]). Dramatic differences in the division mode also occur between different life cycle stages in a single species; asexual stages of *Toxoplasma* in the cat intestine, for example, divide by endodyogeny and endopolygeny [11]. In each case, however, the development will culminate in the emergence of multiple invasive zoites, which seek new host cells to invade. Apicomplexans of the genus *Theileria* are a surprising exception to this divide and conquer scenario. *Theileria* sporozoites remain in the lymphocyte that they initially invade, where they amplify in numbers without resorting to leaving the shelter of the host cell. The key to this trick lies in this parasite's ability to transform the host cell through manipulation of the NFκB pathway. The parasite assembles and activates a mammalian IKK signalosome on its surface, promoting unchecked host cell replication [12,13]. *Theileria* also interacts with host cell microtubules, enabling these parasites to migrate to, and apparently latch onto, host cell centrosomes. This results in partitioning of parasites into forming daughter cells of the host, exploiting the host's mitotic spindle (see Figures 2 and 3; [12,14]; and D. Dobbelaere, personal communication).

## Checkpoints and Master Switches

Initial work using inhibitors of DNA synthesis (e.g., aphidocolin) and microtuble disrupting agents suggested that classical cell cycle checkpoints might be lacking in apicomplexans [15,16], pointing to potentially novel mechanisms of control over their complex cell cycles. However, studies using different blocking agents (thymidine, pyrrolidine dithiocarbamate) and characterization of a series of temperature-sensitive mutants have found that the *Toxoplasma* cell cycle can be halted at what appear to be specific points, including the G1/S and S/M boundaries [17–19]. Furthermore, genomic and experimental surveys for proteins commonly associated with cell cycle checkpoints have identified numerous candidates, including cyclins and cyclin-dependent kinases in *Plasmodium* and *Toxoplasma* [20–23]. An attractive model could suggest the presence of developmentally regulated sets of cell cycle factors resulting in different cell division types, which are in turn controlled by master switches. For example, we could hypothesize that *Toxoplasma* tachyzoites contain master switches to promote nuclear division following DNA synthesis, and cell division following mitosis. Down-regulation of the nuclear division master switch would result in the multiple rounds of DNA synthesis observed during *Sarcocystis* endopolygeny, while down-regulation of the cytokinesis master switch would lead to the multinucleated schizonts observed in other stages of the *Toxoplasma* life cycle and in *Plasmodium* blood stages. Some initial support for this idea has begun to emerge. A series of homologs of the centrosome-associated NIMA kinase (which, in fungi, controls entry into mitosis and spindle formation) have been shown to be essential for cell cycle progression and survival in *Plasmodium* by gene targeting studies [24–27] and in *Toxoplasma* by analysis of temperature-sensitive parasite mutants (M. Gubbels and B. Striepen, unpublished data). NIMA genes appear to be differentially expressed over the *Plasmodium* life cycle. Nek4, for example, is specifically expressed in the female gametocyte and is required for the initial chromosome duplication in the ookinete (zygote) preceding meiosis [25,26], but is dispensable in other stages.

## Counting Chromosomes

**Figure 4:**
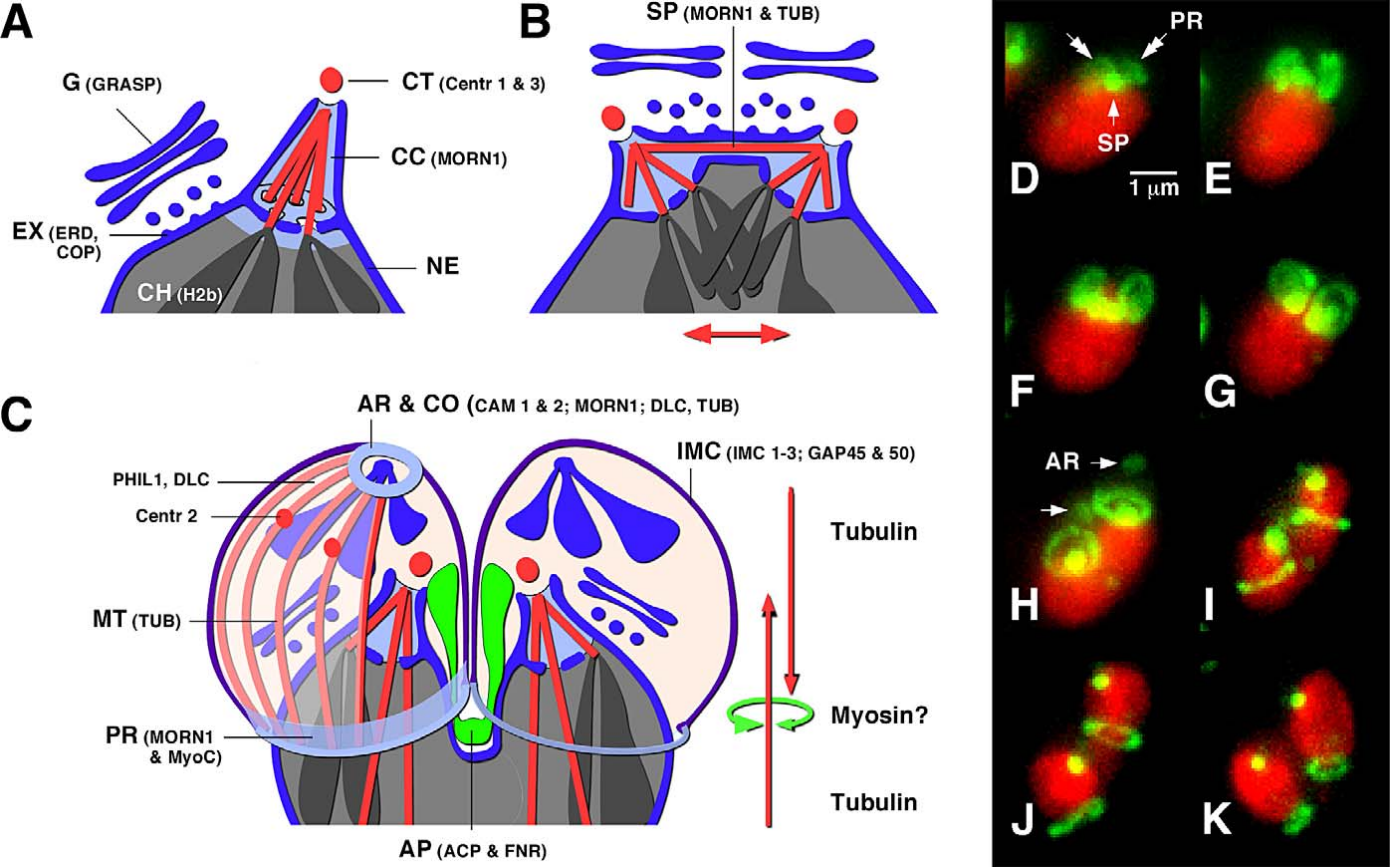
The Mechanics of Apicomplexan Mitosis and Budding (A–C) Schematic representation of the nucleus during interphase (A), mitosis (B), and mid-stage budding (C). Smaller type abbreviations refer to organelle-specific marker proteins in T. gondii (most are available as fluorescent protein in vivo tags, see text for further details and references). AP, apicoplast; AR, apical rings; CC, centrocone; CH, chromosome; CO, conoid; CT, centromere; EX, ER exit site; MT, subpellicular microtubule; NE, nuclear envelope; PR, posterior ring; SP, spindle. (D–K) Time lapse series of nuclear division in T. gondii reproduced from [32]. The nucleus is labeled in red (Histone H2b-RFP) and MORN1 in green (MORN1-YFP).

A fascinating question when considering the various forms of apicomplexan cell division is, how do parasites keep track of their chromosomes in polyploid stages, and how do they know how many zoites to make upon cytokinesis? The following two observations might be important to consider: the final budding of zoites is invariably associated with a last round of DNA replication and nuclear division, and studies that have used high doses of microtubule disrupting agents have found this to lead to a catastrophic breakdown of the coordination of nuclear division and budding in a variety of species [15,28–30]. This suggests that the mitotic spindle, or its organizing center, controls the number of daughter cells and the site where they are to be formed. Apicomplexans use an intranuclear spindle and maintain the nuclear envelope throughout mitosis. The spindle resides in a dedicated elaboration of the nuclear envelope, the centrocone ([31]; Figure 4A), and interacts with the cytoplasmatic centrosome through an opening of the envelope. Interestingly, recent studies in *Toxoplasma* and *Sarcocystis* using antibodies to tubulin and MORN1 (a protein that localizes to the centrocone; see below) have shown that the centrocone is maintained throughout the cell cycle [29,32]. Persistence of the spindle, and persistent kinetochore attachment of chromosomes to the spindle microtubules, would provide a mechanism to maintain the integrity of chromosomal sets through polyploid stages [29]; however, this hypothesis requires experimental validation. While centrocone-like structures have been identified in *Plasmodium* during mitosis and budding [9,33], it is currently not clear if these persist (developing reagents to the *Plasmodium* homolog of the MORN1 protein should quickly resolve this question).

## Building the Zoite Scaffold

Apicomplexans preassemble zoites as buds either internally in the cytoplasm *(Toxoplasma)* or directly under the surface membrane *(Plasmodium).* The scaffold for bud assembly and the outline of the new daughter cells is provided by the pellicle, which consists of subpellicular microtubules and the inner membrane complex (IMC). The subpellicular microtubules emerge from an apical microtubule organizing center associated with the polar rings and run along the longitudinal axis of the cell [34,35]. The IMC is a system of flattened membrane cisternae stabilized by a membrane-associated protein meshwork facing the cytoplasma. Several of the protein components of this meshwork have been characterized and they share weak similarity with articulins, filament proteins found in ciliates [36–38]. Several IMC proteins show dynamic regulation, with their expression timed to coincide with budding [29]. Some IMC proteins also undergo proteolytic processing, and it has been suggested that this process confers increased rigidity to the IMC following its deposition [38,39]. Recently, proteins integral or tightly associated with the outer IMC membrane have been identified. GAP50, together with GAP45, serve as internal anchors of myosin A and the associated gliding motility machinery [40,41]. However, the function of PHIL1, which forms a ring structure at the apical tip of the bud, remains to be elucidated [42].

Following mitotic separation of the chromosomes, budding initiates in the direct vicinity of the centrosomes. The first identifiable sign of the bud is a flattened vesicle associated with a small number of evenly spaced microtubules [8,31,43,44]. This structure is further elaborated into a cup, with the conoid at its apex and microtubules extending from the conoid to posterior ring, delimiting the bud. Genetic and proteomic studies in *Toxoplasma* have identified a number of proteins associated with these early processes, and fluorescent protein tagging and live cell microscopy has painted a highly dynamic picture of their localization and function. The Toxoplasma gondii genome encodes several centrin genes, with centrin 1, 2, and 3 having been localized by GFP fusion [45–47]. While centrin 1 and 3 appear to be focused at the centrosome, centrin 2 additionally labels the conoid and a peculiar group of punctate structures in the apex of the cell [45,46]. Dynein light chain, a component of the minus end-directed microtubular motor dynein, has been detected near the centrosome and the conoid, and may be involved in conoid and centrosomal movements. MORN1 has been particularly informative as a marker for budding, as it labels both the centrocone and spindle and the apical and posterior ends of the bud (Figure 4; [32,46]). The precise chronology of assembly—especially in the very early phase of bud development—remains to be elucidated, and would benefit from the generation of mutants for the various steps involved. Early electron microscopic studies have implicated a striated fiber as an organizing element [44]; interestingly, proteins similar to algal-striated fiber assemblins have been identified recently in apicomplexans and have been shown to localize to the centrosomal region during budding [48]. Once the bud is assembled it grows rapidly, most likely driven by microtubule growth. This process runs opposite to spindle extension and effectively partitions the nucleus and much of the cytoplasm. Toward the end of bud development, the MORN1 ring at the posterior end of the bud shows pronounced contraction (see Figure 4J and 4K), which likely aids in organellar division (see below) and cytokinesis. Several observations are consistent with an association of this ring with myosin B/C [32,49]; however, the actin-destabilizing drug cytochalasin D does not interfere with parasite division [15].

## Completing Parasite Assembly

A fully formed apicomplexan parasite requires a multitude of organelles and intracellular structures that will enable it to carry out the next task of its life cycle—to egress from the host cell and invade a new one. Rhoptries, micronemes, and dense granules form de novo during budding, anterior to the nucleus, endowing each daughter cell with the apical secretory organelles necessary for invasion. Expression of rhoptry and microneme proteins is regulated at the transcriptional level and timed to conincide with budding [50–53]. The apicomplexan secretory pathway is highly polarized, with an endoplasmic reticulum (ER) exit site localized on the apical face of the nucleus adjacent to the centrocone [43,54,55]. Here, proteins are loaded into coated vesicles that travel to the Golgi and on to several (still poorly characterized) trans-Golgi, pre-rhoptry, and pre-miconeme compartments [54,56,57]. The Golgi is associated with the centrosome(s), which play an important role in its duplication [58]. Golgi duplication is among the earliest events of budding [47,59]. In *Plasmodium,* the Golgi divides multiple times during intracellular development, and upon zoite formation, a single Golgi is associated with each bud [60]. The spatially fixed line-up of ER exit site and Golgi and their association with the nucleus and centrosome likely acts as a highly effective cellular “funnel”, directing the flow of proteins and membranes into the growing buds. IMC proteins, including the N-glycosylated GAP50 [40], probably derive from the Golgi, suggesting that membranes of the IMC form from Golgi-derived vesicles. This would explain the necessity for early division of the Golgi during budding, and suggests that Golgi positioning by the centrosome is critical in mediating deposition of the IMC.

**Table 1:**
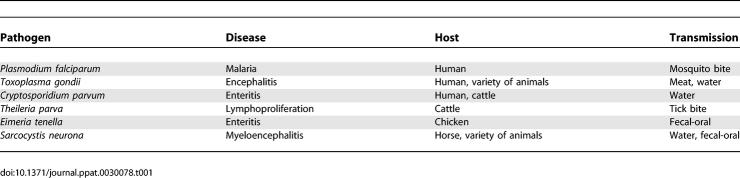
Apicomplexan Parasites

Apicomplexans harbor two endosymbiont-derived organelles, the mitochondrion and the apicoplast, both of which perform a broad array of metabolic functions and are essential for intracellular parasite development [61–64]. These organelles carry their own genomes [65–69] and therefore can not be formed de novo, but must undergo division followed by segregation into buds. Genomic analyses in apicomplexans have identified proteins commonly involved in mitochondrial division, like dynamin-related proteins [62]. However, the FtsZ-based division machine found in a wide variety of chloroplasts has been lost in apicomplexans [70,71]. Instead of relying on their ancestral prokaryotic division ring, it would appear that apicoplasts have developed novel means of division. One model suggests that the force for apicoplast division is provided by association of the apicoplast with the mitotic spindle [72]. Dynamic association between the centrosome(s) and the apicoplast has been demonstrated in *Toxoplasma* and *Sarcocystis* and provides a likely means by which these organelles are properly segregated into forming buds [29,72]. In both organisms, fission of the organelle into daughter plastids is tightly associated with budding, and the constrictive MORN1 ring found at the posterior end of each bud provides an attractive candidiate for a fission mechanism (see Figure 4C; [32,71]). A second model suggests that apicoplast fission is independent of cytokinesis and relies on a medial division ring formed by yet-to-be identified components [73]. The development of the plastid in organisms dividing by schizogony, like *Plasmodium* and *Eimeria,* is not fully understood [74,75]. While centrosome association is likely to be involved in the segregation into daughters, it is unclear if such association occurs in earlier stages. In *Plasmodium,* mitochondria and apicoplasts form a physical association shortly before budding [74], suggesting that segregation of these organelles into daughter buds is tightly linked. Nevertheless, better in vivo markers (especially for the centrosome) are needed to identify mechanisms of organellar division and segregation in these organisms.

## Outlook

The advent of reverse genetics for a variety of apicomplexans has led to a renaissance in the study of the cell biology of these parasites. A number of exciting new structures and mechanisms have been discovered in this process. Not unlike the study of host cell invasion, exploring the intracellular development of apicomplexans has brought out conserved themes at the mechanistic level, suggesting significant similarity between different species within the phylum. The “post-genomic” era of apicomplexan cell biology offers powerful experimental avenues that will undoubtedly drive our understanding of cell division and zoite formation. Gene expression profiling using microarrays, now available for several systems, has identified large groups of candidate genes that are expressed during budding. Comparative genomic analysis can be used to further narrow the list of candidates. The ever improving forward and reverse genetics tool box offers robust experimental avenues to test the function of essential genes, and genetic analysis will be critical for establishing the sequence of events during budding [76]. The coming years will likely reveal an increasingly detailed and mechanistic picture of these tiny diabolical, yet fascinating, invasion machines. 

## Abbreviation

IMC: inner membrane complex
